# Our New York Letter, Bone Surgery

**Published:** 1885-12

**Authors:** Wm. Penny


### OUR NEW YORK LETTER.

### BONE SURGERY IN N. Y. HOSPITALS.

Editor Daniel's Texas Medical Journal:

*Dear Sir:*

I PROMISED to give you an account of the practice of gynecological surgery, in this medical center; but I will have to crave an extension of time, as I have not filled myself up as yet. I will, however, give your readers a few points on the surgery of the bones and joints.

The discussion on the subject of the etiological factor in joint diseases still waxes hot and the followers of Sayre, battle for his theory of traumatism, while the opposing host point to the bacilli found in carious bone as conclusive proof that the disease is tuberculous in its origin. Neither side has convinced the other as yet, and as a result we have as great a diversity in the treatment of joint diseases as in the the theories as to their cause.

The orthopœdists, as a rule, including members of both factions, hold the joints in the same awe that the surgeon held the peritoneum a couple of decades since. While the general surgeon, having sworn on the great book of science to exterminate this micro-organism wherever found, and to find it wherever it may be hid, have no hesitation in opening the joints, and by cutting, scraping and gouging to get rid of all diseased bone and tissues. The theory is most excellent, and appeals strongly to the large classes of students that witness these surgical feats. I have witnessed quite a number of these resections, (they are all classed as resections), and while the work was thorough,and skilfully done, with all the aid of antiseptics and skilled assistants, the average result has not been a brilliant success. The diseased bone necessarily left the tissues infiltrated with inflamatory products; or the microbe, or all of them combined, set up an acute inflammation, and the end is worse than the beginning. Not all of these cases terminate this way, for in some, there are brilliant results, but they are the exceptions and not the rule.

It is quite amusing sometimes to hear one of these hospital surgeons say to a class of students, "Gentlemen, if I can streighten this limb without two much force, I will put on an immovable dressing and trust this knee to nature." While he knows all the time that he cannot streighten the limb without doing irreparable injury to the joint. Of course he does not streighten the limb, and as a consequence he resects. As the boys say, his mouth was watering for the resection all the time. It does look, after having seen a number of these joints opened and diseased bone found every time, that the heroic plan would be the best, but these joints affected with carious bone and undergoing a chronic inflammatory process are not tolerant of surgical interference. The orthopœdist makes use of some form of fixative apparatus, and waits;—waits usually a long time, and then gets an anchylosed limb with more or less deformity, and considerable practical shortening.

They claim that the mortality is notably less than under the heroic plan of treatment. Statistics, however, are hard to get in these cases, as they change from one surgeon to another, and the one who gets the case first rarely sees the end of it.

Upon the subject of sinuses leading to dead bone, there is no difference of opinion. The rule is: Open up the sinus, gouge out the dead bone and detritus, scrape the sinus and close the wound, after putting in a drainage tube. In necrosis, the old rule of waiting for a sequestrum to form is not followed: the periosteum is elevated, the dead bone removed, and the wound closed, antiseptically, of course.

As an example of the daring manner in which surgeons follow up sinuses, I saw Dr. Wyeth open up some sinuses situated half way between the knee and ankle, until he found the carious bone in the lower part of the femur, in front of the vessels and nerves in the popliteal space; he then scraped the carious bone and finished his operation.

Before closing, I will give you another novelty in surgery; it is called canalization. It is a great favorite of Dr. Gerster, whom I saw perform it in a case of necrosis of the tibia. In this case, the necrosis extended nearly the entire length of the tibia. After the incision and removal of the diseased bone, the flaps were turned in, and pegged to the bone with small iron nails, such as are used in the manufacture of cigar boxes; then a piece of rubber protective was laid in the trough thus made; next, the wound was packed with antiseptic gause, and the external dressing applied. By this means the wound was converted into a canal or gutter, requiring no drainage tubes. It will be seen that this is carrying out the principle of Markoe's open drainage, and that of the antiseptic plan at the same time. It is, however, German.

If I had the space, I would like to take up the operations on the separate joints, but that would require a book and not a letter.

WM. PENNY, M. D.

